# Functional diversity of the *Osiris* gene family in the brown planthopper

**DOI:** 10.1007/s44297-025-00045-4

**Published:** 2025-03-12

**Authors:** Cui Zhang, Xinyi He, Ya Ma, Yaxin Liu, Xingxing Shen, Yanyuan Bao

**Affiliations:** 1 Institute of Insect Sciences, Ministry of Agriculture and Rural Affairs Key Laboratory of Molecular Biology of Crop Pathogens and Insect Pests, College of Agriculture and Biotechnology, Zhejiang University, Hangzhou, China; 2 Zhejiang Key Laboratory of Biology and Ecological Regulation of Crop Pathogens and Insects, Zhejiang University, Hangzhou, China; 3 Department of Integrated Biosciences, Graduate School of Frontier Sciences, The University of Tokyo, Chiba, Japan

**Keywords:** *Nilaparvata lugens*, *Osiris* genes, RNA interference, Molecular function

## Abstract

*Osiris* is a large insect-specific gene family with multiple functions. Owing to highly conserved sequences and similar expression patterns across insect species, the *Osiris* gene family is believed to play specific roles in the insect kingdom. However, current studies on *Osiris* gene functions have focused primarily on several holometabolous insects, such as *Drosophila melanogaster* and *Bombyx mori*, with few systematic studies on hemimetabolous insects. In this study, we identified 20 *Osiris* genes by searching the genome and transcriptome databases of the brown planthopper *Nilaparvata lugens*. The *Nilaparvata lugens Osiris* genes presented similar development- and tissue-specific expression profiles. A functional investigation using RNAi in 4th instar nymphs and newly eclosed adult females revealed that the knockdown of *Osiris* genes resulted in multiple phenotypes, including a lethal phenotype; morphological changes in the wing, ovary and ovipositor; and hatching and feeding failure. These observations suggested that the *Osiris* genes play different functional roles at the individual and tissue levels. This study expanded the knowledge of *Osiris* gene functions and provided a better understanding of the importance of the *Osiris* gene family in the development and reproduction of insects.

**Supplementary Information:**

The online version contains supplementary material available at 10.1007/s44297-025-00045-4.

## Introduction

The *Osiris* gene family was first described in *Drosophila melanogaster* [1]*.* Twenty-five *Osiris* genes have been identified in the Drosophila genome (FlyBase) [2]. Among these genes, 22 clustered within the dosage‐sensitive Triplo-lethal (*Tpl*) locus in the chromosomal region 83D-E. The other three genes (*Osiris21*, *22* and *23*) are outside of this cluster and are located at the 32E, 99F and 87E sites in the genome [1]. The Osiris proteins are characterized by a DUF1676 domain (Pfam: PF07898) with unknown function [3]. In addition, they feature an N-terminal signal peptide, a pair of conserved cysteine residues, a transmembrane region and a C-terminal AQXLAY motif [4].

The *Osiris* genes are present only in insects, from the basal groups of mayflies and silverfish to highly evolved dipterans [4–6]. No obvious homologs have been reported in the genomes of non-insect Arthropoda or other invertebrates, including crustaceans, Myriapod, Chelicerata, and Entognatha (Collembola) [6–8]. A few studies, primarily in the fruit fly *D. melanogaster* and the silkworm *B. mori*, have addressed the specific functions of the *Osiris* genes [7–11]. In *D. melanogaster*, of the 25 *Osiris* genes, 16 were expressed in cuticle-secreting epidermal and sensory organ cells, and 4 were required for specific cuticle nanostructures [6]. The embryonic lethal *Osiris6* and *Osiris7* mutants presented strong defects in larval cuticle formation [7]. *Osiris6*, *Osiris7* and *Osiris8* are involved in resistance to octanoic acid (OA) [10, 12–14], a remarkable plant toxin to Drosophila species, with the exception of *Drosophila sechellia*, which is a dietary specialist on the host plant *Morinda citrifolia* and has evolved resistance to fruit OA [10]. *Osiris8* is highly expressed in the tormogen support cells of the antenna trichoid sensilla and is required for pheromone detection [9]. *Osiris9*, *Osiris15* and *Osiris19* are highly expressed in the trachea and function redundantly to regulate tracheal tube maturation [8]. *Osiris17* is required in the wing epithelium to produce expanded wings [6]. *Osiris21* is involved in endolysosomal trafficking in photoreceptor neurons of the eye [15], suggesting that it plays a role in regulating the cellular signaling pathways involved in vesicular trafficking and protein sorting within this system. *Osiris23* is essential for the formation of nanopores lining the olfactory sensillum [7]. In *B. mori*, 25 *Osiris* genes have been identified, and most of these genes are specifically expressed in the wings or epidermis [16]. In particular, the *Osiris9a* gene in the silkworm *Osiris* family is specifically expressed in the silk gland and contributes to the formation of silk fibers [17]. The understanding of the functions of *Osiris*, an insect-specific gene family, has been limited in the above two model insect species. Although a recent study reported that *Osiris17* contributes to morphogenesis of the intestinal tract in the hemimetabolous insect *Locusta migratoria* [18], the physiological functions of this gene family in most insect species are still largely unknown. Although some genes with functions of both chitin-binding and peptidase activity are coexpressed with the *Osiris* family [11], the precise interaction mechanism remains elusive. To better understand the functional roles of the *Osiris* gene family, in this study, we aimed to study a Hemipteran insect, the brown planthopper (*Nilaparvata lugens* Stål), to conduct a comprehensive analysis of the expression and functions of the *Osiris* genes. *Nilaparvata lugens* is an important hemimetabolous rice pest that causes severe damage to rice by sucking rice phloem sap [19]. This insect species has emerged as the ideal model system for studying gene functions because the whole-genome sequence has been elucidated [20], and the susceptibility to RNA interference (RNAi) [21–26].

In this study, we identified 20 *Osiris* family genes by searching the *N. lugens* genome and transcriptome databases and analyzed their phylogenetic relationships, spatiotemporal expression patterns, and gene functions via RNA interference (RNAi). The knockdown of the *Osiris* genes resulted in various phenotypes, including lethality, wing and ovipositor deficiency, hatching and feeding failure. To our knowledge, this is the first report on the reproductive and feeding functions of *Osiris* genes. Our findings increase the understanding of the physiological functions of *Osiris* genes in development and reproduction. This gene family could be a potential target for pest management to develop biological control strategies involving genetic modification to reduce insecticide usage.

## Materials and methods

### Insects and plants

Brown planthopper populations were originally collected from a rice field located at the Huajiachi Campus of Zhejiang University, Hangzhou, China, in 2008. The insect strain was routinely reared on rice seedlings (*Oryza sativa* strain TN1) at a temperature of 26 ± 0.5 °C with 50 ± 5% relative humidity under a 16:8 h light:dark photoperiod as previously described [21].

### Identification of the *N. lugensOsiris* gene family

The *Osiris* gene sequences were obtained from the *N. lugens* genome (GenBank accession number AOSB00000000 under BioProject PRJNA177647) and transcriptome (accession number SRX023419) databases via the BLASTX algorithm with a significance cutoff of E value < 1×10^−5^. The *Osiris* sequences were further verified and annotated via the National Center for Biotechnology Information (NCBI), Conserved Domains Database (CDD) (https://www.ncbi.nlm.nih.gov/Structure/cdd/cdd.shtml) and SwissProt database (https://www.sib.swiss/swiss-prot). The open reading frames (ORFs) and amino acid sequences were predicted via Open Reading Frame Finder (https://www.ncbi.nlm.nih.gov/orffinder/). The signal peptide and characteristic domain were predicted via the Simple Modular Architecture Research Tool (SMART) (https://smart.embl.de/).

### Phylogenetic tree construction

To understand the phylogenetic relationship, the *Osiris* sequences were obtained from 23 insect species in 8 orders, including Odonata: *Ischnura elegans*; Orthoptera: *Schistocerca americana*; Hemiptera: *Laodelphax striatellus*, *N. lugens*, *Acyrthosiphon pisum*, *Rhopalosiphum maidis*; Hymenoptera: *Nasonia vitripennis*, *Vespula pensylvanica*, *Apis mellifera*, *Ooceraea biroi*; Coleoptera: *Agriotes lineatus*, *Tribolium castaneum*, *Diorhabda carinulata*, *Brassicogethes aeneus*; Lepidoptera: *Pararge aegeria*, *Pectinophora gossypiella*, *Trichoplusia ni*, *B. mori*; Neuroptera: *Chrysoperla carnea*; Diptera: *D. melanogaster*, *Anopheles merus*, *Aedes aegypti*, *Hermetia illucens*. The *Osiris* sequences of these insect species were downloaded from the NCBI website (https://www.ncbi.nlm.nih.gov/), FlyBase Drosophila database (https://flybase.org/) and KAIKO (https://kaikobase.dna.affrc.go.jp/) by using *D. melanogaster* and *B. mori Osiris* sequences as queries to search the homologs of the other insect species via BLAST v2.12.0 (http://ftp.ncbi.nlm.nih.gov/blast/executables/blast+/2.12.0) at an E-value threshold of 1×10^−5^. The predicted *Osiris* genes were filtered on the basis of an identity threshold of ≥ 30%. InterProScan v5.66 (https://ftp.ebi.ac.uk/pub/software/unix/iprscan/5/5.66-98.0/interproscan-5.66-98.0-64-bit.tar.gz) [27] was used to determine the reliability of the homologous Osiris proteins across different insect species. Given the intrinsic homology among genes within the *Osiris* family, we employed IQ-TREE v2.3.3 (http://iqtree.cibiv.univie.ac.at/) to construct a maximum likelihood (ML) tree of the candidate Osiris protein sequence alignment under the Q. insect + F + R10 model [28, 29]. The reliability of the ML phylogeny was assessed via a bootstrap analysis of 1000 replications; bootstrap values N50% are shown on each node of the tree. The phylogenetic tree was visualized via iTOL v5 (https://itol.embl.de) [28].

### Spatiotemporal expression patterns of *NlOsiris* genes

The spatiotemporal expression profiles of *NlOsiris* genes throughout the developmental stages and in various tissues were investigated by searching the *N. lugens* transcriptomic database (GenBank accession no. PRJNA714229) as described previously [30–32]. To verify the expression profiles, quantitative real-time PCR (qRT‒PCR) was conducted. Total RNA was extracted from each developmental stage and each tissue via a TRIzol Total RNA Isolation Kit (TaKaRa, Dalian) according to the manufacturer's instructions. The RNA concentrations were determined via a NanoDrop 2000 Spectrophotometer (Thermo Fisher Scientific). For developmental stage-specific expression analysis, total RNA was extracted from the eggs in the rice leaf sheaths at 24, 72 and 144 h (*n* = 100) after laying; the whole bodies of the 1st-instar nymphs at 0 and 36 h after hatching (*n* = 100); the 2nd-instar nymphs at 0 and 36 h after molting (n = 80); the 3rd-instar nymphs at 0 and 36 h (*n* = 50); the 4th-instar nymphs at 0, 12, 24, 36, 48 and 60 h (*n* = 40); the 5th-instar female nymphs at 0, 12, 24, 36, 48, 60 and 72 h (*n* = 40); the 5th-instar male nymphs at 0 and 48 h (*n* = 40); the female adults at 0, 12, 24, 36, 48, 60 and 72 h (*n* = 25); and the male adults at 0, 24 and 48 h after eclosion (*n* = 25). For tissue-specific expression analysis, total RNA was extracted from the fat body (*n* = 100), integument (*n* = 80), wing bud (*n* = 80), gut (*n* = 100), and salivary gland (*n* = 200) of 5th-instar nymphs spanning early to late developmental stages and from the ovary (*n* = 50) and testis (*n* = 50) of female and male adults at 24–72 h post-eclosion. Prior to dissection, insects were anesthetized on ice and carefully dissected under a Leica S8AP0 stereomicroscope (Leica Microsystems GmbH, Wetzlar, Germany) with fine forceps. The isolated tissues were gently washed in phosphate-buffered saline (PBS) for RNA extraction. The total RNA of each sample served as a template for reverse transcription via a Hiscript® II QRT SuperMix for qPCR (+ gDNA wiper) Kit (Vazyme, Nanjing, China) to remove any contaminating genomic DNA. qRT‒PCR was performed on a CFX ConnectTM Real-Time System (Bio-Rad, Hercules, CA, USA) using ChamQ SYBR Color qPCR Master Mix (Vazyme, Nanjing, China) under the following reaction program: an initial denaturation step at 95 °C for 30 s, followed by 35 cycles at 95 °C for 5 s and 55 °C for 30 s. For each *NlOsiris* gene, specific primers for qRT‒PCR were designed via Primer Premier 6 (Premier, Biosoft, Canada) to amplify regions ranging from 150–250 bp (Table S1). The *18S ribosomal RNA* gene (GenBank accession number JN662398.1) was used as an internal control. Relative expression levels were calculated via the 2^−ΔΔCt^ method [33]. Each sample was analyzed in triplicate. Developmental and tissue-specific expression heatmaps were generated via GraphPad Prism 8 (La Jolla, CA, USA).

### RNA interference (RNAi)

RNAi was used to investigate the functions of the *Osiris* gene family in *N. lugens*. Each *NlOsiris* gene was amplified and cloned and inserted into the pMD-19 T vector (TaKaRa, Dalian, China). Double-stranded RNAs (dsRNAs) were synthesized with specific primers containing a T7 promoter in vitro through PCR-generated DNA templates via a T7 High Yield RNA Transcription Kit (Vazyme, Nanjing, China). The sequences of the *NlOsiris* genes used as templates for dsRNA synthesis ranged from 550–650 bp in length. ds*GFP* served as a negative control to assess nonspecific effects. The specific primers used for dsRNA synthesis are shown in Table S1.

For gene function analysis, the nymphs were anesthetized with carbon dioxide for 20 s, and then 10 µL of each dsRNA (5 ng/µL) was microinjected into the abdomen of 4th-instar nymphs (24–48 h after molting, *n* = 100) via a FemtoJet microinjection system (Eppendorf-Netheler-Hinz, Hamburg, Germany) [21]. For each treatment, three biological replicates were conducted. RNAi efficiency was assessed in 5th-instar nymphs at 48 h after molting. Phenotypes, including survival and any abnormally morphological phenotypes, were recorded daily. For reproduction studies, each newly emerged adult (0–2 h post-eclosion, n = 50) was anesthetized with carbon dioxide for 25 s and microinjected with 15 µL of each dsRNA (5 ng/µL). After injection, the insects were reared on fresh rice seedlings. Mating between one female and one male was performed 3 days after RNAi, and the females were removed from the rice seedlings on the 5th day after mating. The number of hatched nymphs was counted daily for 7 days. The seedlings were dissected to observe and calculate the unhatched fertilized and unfertilized eggs. RNAi efficiency in the eggs was assessed at 144 h post-laying within the rice seedlings via qRT‒PCR. Data analysis was performed via GraphPad Prism 8.0 (La Jolla, CA, USA). The data were analyzed via Student's *t* test, and the results are presented as the means ± standard deviations.

### Feeding observation

The artificial D-97 diet was prepared according to methods described by Zu et al. [34]. The artificial diet was prepared under aseptic conditions, filtered through a 0.22 μm syringe filter (Millipore, MA, USA), and stored at -20℃. The 4th-instar nymphs were injected with ds*Osiris* RNAs and reared on fresh rice seedlings. On the 3rd day postinjection, the nymphs were transferred from the rice seedlings to glass tubes (length: 8 cm, diameter: 4.5 cm) and reared for 7 days. Both ends of a glass tube were sealed with two layers of Parafilm membranes (Pechiney Plastic Packaging Company, Chicago, IL, USA) with 40 μL of artificial diet in the space between the two layers of Parafilm. The glass tubes were placed at 26℃ ± 5℃, with a relative humidity of 50% ± 5% and a photoperiod of 16 h light and 8 h dark. Each glass tube contained 15 nymphs, with four biological replicates per treatment. The artificial diet was replaced every 24 h, and the number of surviving insects was calculated. The salivary sheaths were carefully separated from the inner layer of Parafilm via forceps under a stereomicroscope (Leica S8AP0, Wetzlar, Germany). The number of salivary sheaths was recorded. The data were analyzed via Student's *t* test, and the results are presented as the means ± standard deviations.

## Results

### Phylogenetic analysis of NlOsiris proteins

**Fig. 1:**
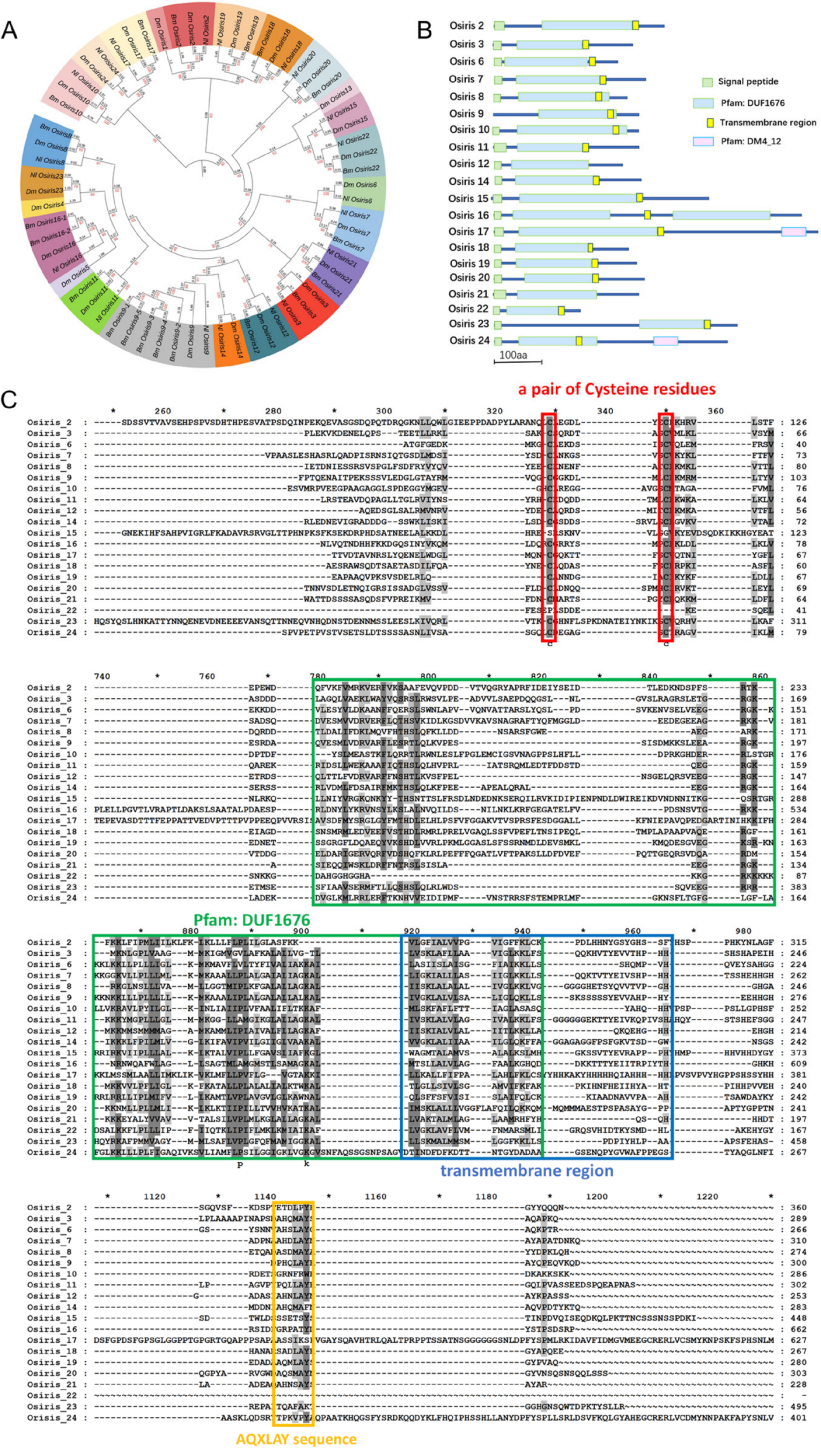
Bioinformatics analysis of the putative NlOsiris proteins*.* **A** Phylogenetic tree of insect Osiris proteins. A phylogenetic tree was constructed on the basis of the amino acid sequences of insect Osiris proteins via MEGA X (http://www.megasoftware.net/) with the maximum likelihood method with 1000 bootstrap replicates. Dm, *D. melanogaster*; Bm, *B. mori*; Nl, *N. lugens.* **B** Prediction of NlOsiris protein structure. The conserved domains of NlOsiris proteins were determined via SMART (http://smart.embl.de/), Pfam (http://pfam.xfam.org/) and NCBI (http://www.ncbi.nlm). The blue bars represent the number of amino acid residues, while the colored boxes indicate the characteristic domains. **C** Multiple sequence alignment of NlOsiris proteins. The conserved sequences, including a pair of conserved cysteine residues, the DUF1676 domain, the transmembrane region and the AQXLAY sequence, are highlighted in colored boxes

We identified 20 *Osiris* genes in *N. lugens* by searching genomic and transcriptomic databases (Table 1). The encoded proteins were validated through protein domain prediction (with a score < 0.001) (Table S2). We constructed a phylogenetic tree based on the complete amino acid sequences available in the GenBank database to understand the evolutionary relationships among NlOsiris and their homologs in other insect species. Phylogenetic analysis indicated that each NlOsiris protein is closely related to *D. melanogaster* and *B. mori* homologs (Fig. 1A). We follow the nomenclature strategy of *D. melanogaster* and *B. mori Osiris* genes to name the *N. lugens* homologs *NlOsiris2*-*3*, *NlOsiris6*-*12* and *NlOsiris14*-*24* on the basis of the the best match of the amino acid sequences with *D. melanogaster* and *B. mori* Osiris proteins. Although the E value of NlOsiris15 was > 1×10^−5^ according to the BLASTp algorithm, its evolutionary relationship suggests that it is a member of the Osiris family (Table S2).

**Table 1:** Identification of *Osiris* genes in the *N. lugens* genome

Name	Genomic location	Size (aa)	Orientation	Domains	Best match	E-value
*osiris 2*	chr1[71781106–71785372]	360	-	Duf1676	D. *melanogaster*	1.00E-33
*osiris 3*	chr1[16144454–16155056]	289	+	Duf1676	*B. mori*	2.00E-38
*osiris 6*	chr1[98218969–98225006]	266	-	Duf1676	*D. melanogaster*	1.00E-25
*osiris 7*	chr1[21455532–21466129]	310	-	Duf1676	*D. melanogaster*	5.00E-34
*osiris 8*	chr1[21416194–21423999]	274	-	Duf1676	*D. melanogaster*	2.00E-29
*osiris 9*	chr1[21356327–21365789]	300	-	Duf1676	*B. mori*	2.00E-27
*osiris 10*	chr1[78034913–78038815]	286	-	Duf1676	*B. mori*	2.00E-14
*osiris 11*	chr1[77944874–77949318]	292	-	Duf1676	*D. melanogaster*	1.00E-36
*osiris 12*	chr1[77897261–77910238]	253	-	Duf1676	*D. melanogaster*	5.00E-20
*osiris 14*	chr1[77830153–77837895]	283	-	Duf1676	*D. melanogaster*	6.00E-42
*osiris 15*	chr1[77812888–77815139]	448	-	Duf1676	*D. melanogaster*	/
*osiris 16*	chr1[77763152–77782145]	662	-	Duf1676	*B. mori*	1.00E-08
*osiris 17*	chr1[72092224–72117547]	680	+	Duf1676, DM4_12	*D. melanogaster*	7.00E-28
*osiris 18*	chr1[86484346–86489074]	267	-	Duf1676	*B. mori*	3.00E-39
*osiris 19*	chr1[90906218–90909732]	280	-	Duf1676	*B. mori*	2.00E-61
*osiris 20*	chr1[90888853–90893016]	303	-	Duf1676	*D. melanogaster*	1.00E-26
*osiris 21*	chr3[14683004–14688778]	228	+	Duf1676	*B. mori*	2.00E-14
*osiris 22*	chrX[2469205–2491639]	179	-	Duf1676	*B. mori*	4.00E-08
*osiris 23*	chr8[36186068–36194529]	495	-	Duf1676	*D. melanogaster*	3.00E-06
*osiris 24*	chr1[90769914–90778891]	478	+	Duf1676, DM4_12	*D. melanogaster*	3.00E-54

The *Osiris* sequences were obtained from the *N. lugens* genomic and transcriptomic databases. The *Osiris* genes were confirmed via the BLASTp algorithm with a cut-off E value of 1×10^−5^. The location, size and orientation indicate the locus, predicted amino acids (aa) and transcription orientation of the genes on the chromosome. Domains refer to the presence of the characteristic Duf1674 domain and/or the transmembrane domain DM4_12. *D. melanogaster*, *Drosophila melanogaster*; *B. mori*, *Bombyx mori*

As the synteny of the *Osiris* genes was presented in the *D. melanogaster* and *A. gambiae* genomes, we examined the location of the *Osiris* genes in the *N. lugens* genome. Among the 20 *NlOsiris* genes, 17 were located on chromosome 1, whereas *NlOsiris21*, *NlOsiris22*, and *NlOsiris23* were located on chromosomes 3, X, and 8, respectively. We still mapped the chromosomal locations of the *Osiris* genes across 23 insect species, and synteny was detected in the genomes of all 23 insect species (Figure S1). The predicted NlOsiris proteins had varying sizes, consisting of 179–680 amino acids (Table 1). All NlOsiris proteins contained a characteristic Duf1676 domain (Pfam: PF07898) (Fig. 1B). NlOsiris16 is special because it has two Duf1676 domains. The NlOsiris proteins had a predicted signal peptide at the N-terminus and a transmembrane region, except for NlOsiris9, which lacks a signal peptide sequence, and NlOsiris21, which lacks a transmembrane region. Additionally, NlOsiris17 and NlOsiris24 each contained a function-unknown Dm4_12 domain (Pfam: PF07841), a 115-aa motif with four highly conserved cysteine residues near the C-terminus. Multiple sequence alignments of NlOsiris proteins revealed conserved motifs, including a pair of conserved cysteine residues near the N-terminus, the Duf1676 domain, the transmembrane region and an AQXLAY sequence near the C-terminus (Fig. 1C).

### Spatiotemporal expression analysis of *NlOsiris* genes

**Fig. 2:**
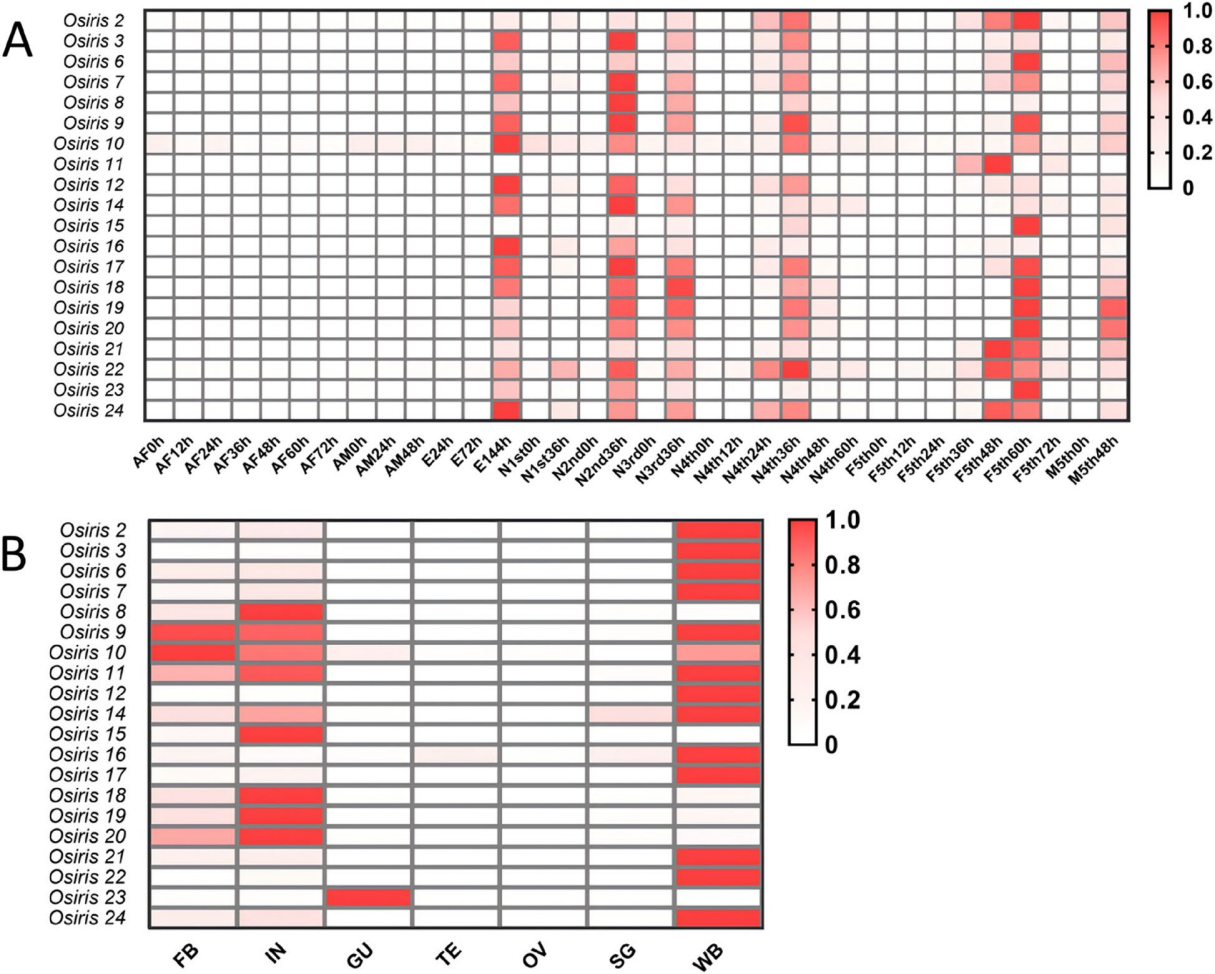
Heatmap of the spatiotemporal expression profile of *NlOsiris* genes. **A** Developmental expression profiles of *NlOsiris* genes were obtained from the transcriptome data and visualized as a heatmap with GraphPad. The y-axis represents *NlOsiris* genes, and the x-axis denotes different developmental stages. AF, adult female; AM, adult male; E, egg; N, nymph; F5th, fifth-instar female nymph; M5th, fifth-instar male nymph. **B** Tissue-specific expression profiles of *NlOsiris* genes were obtained via qRT‒PCR and visualized as a heatmap with GraphPad. The y-axis represents *NlOsiris* genes, and the x-axis denotes the different tissues. FB, fat body; IN, integument; GU, gut; SG, salivary glands; WB, wing buds, which were dissected from fifth-instar nymphs. TE, testis; OV, ovary, dissected from male and female adults, respectively. Red indicates relatively high expression levels, and white represents relatively low expression levels

To understand the functions of the *Osiris* genes in *N. lugens*, we investigated their development- and tissue-specific expression patterns by searching the *N. lugens* transcriptome database and confirmed the development- and tissue-specific expression profiles of the *NlOsiris* genes via qRT‒PCR (Fig. 2A). The *NlOsiris* transcripts were almost undetectable or present at extremely low levels in female and male adults. For the eggs laid in the rice leaf sheaths, almost no transcripts were detected in the eggs at 24 and 72 h post-laying, but they were detected at high levels at 144 h, a time point before egg hatching. Notably, the *NlOsiris* genes presented periodic expression patterns throughout the nymphal stages. The transcripts were not detectable or at extremely low levels in newly hatched 1st–5th-instar nymphs at 0 h and increased to reach peak levels at the middle stage of each instar nymph, namely, at 36 h for the 1st–4th-instar nymphs and 48–60 h for the 5th-instar female and male nymphs. These results suggest that *NlOsiris* genes may function in egg hatching and the development of nymphs. Among the *NlOsiris* genes, *NlOsiris11* presented a different expression pattern, whose transcripts were exclusively detected in 5th-instar female nymphs, with expression peaking at 36–48 h, implying a special function at the developmental stage.

The *NlOsiris* genes presented tissue-specific expression patterns (Fig. 2B). The transcripts of most *NlOsiris* genes were detected at high levels in the wing bud, integument and/or fat body but were not detectable or at extremely low levels in the gut, testis, ovary and salivary gland. Despite the similar expression patterns, differences could be observed in these genes. *NlOsiris8*, *NlOsiris15*, *NlOsiris18*, *NlOsiris19* and *NlOsiris20* transcripts were detected at very high levels in the integument followed by the fat body but were not detected in the wing bud. The *NlOsiris12* transcript was detected only in the wing bud, and the *NlOsiris23* transcript was detected only in the gut. *NlOsiris9*, *NlOsiris10* and *NlOsiris11* transcripts were detected at similarly high levels in the wing bud, integument and fat body. The transcripts of the other *NlOsiris* genes, including *NlOsiris2, NlOsiris3, NlOsiris6, NlOsiris7, NlOsiris14, NlOsiris16, NlOsiris17, NlOsiris21, NlOsiris22 and NlOsiris24,* were detected at high levels in the wing bud but at low levels in the integument and fat body.

### Effects of *NlOsiris* gene knockdown on development

**Fig. 3:**
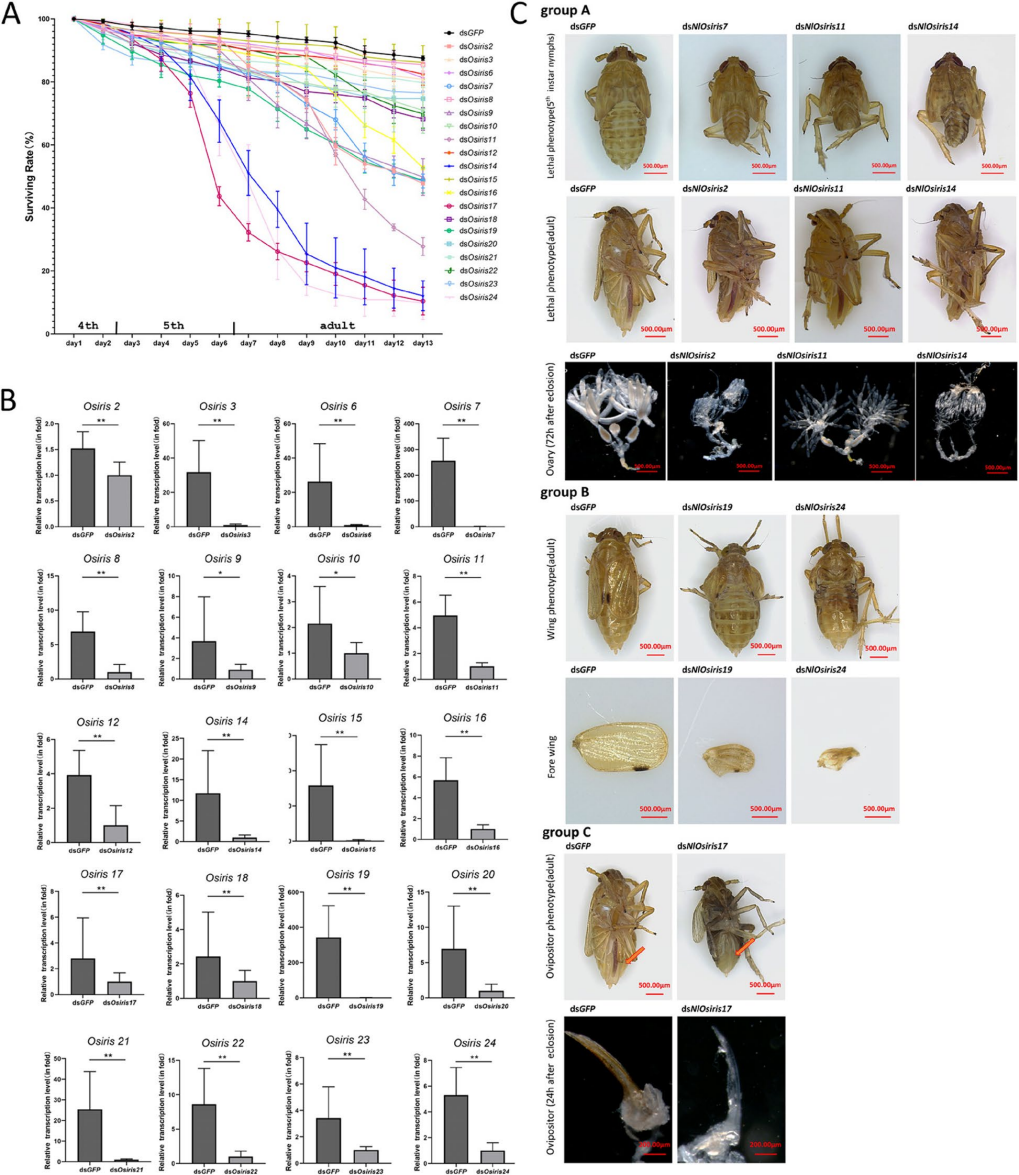
Effects of *NlOsiris* gene knockdown on development. **A** Dynamic analysis of the survival rates of *N. lugens.* The 4th-instar nymphs were injected with ds*NlOsiris* (50 ng per insect) and observed for phenotypic variation at 24 h intervals for 13 days. ds*GFP* was injected as a negative control to determine the non-specific effects of dsRNA. Three independent biological replicates were conducted for each treatment (mean ± standard deviation; *n* = 80–100 nymphs). **B** Determination of RNAi efficiency. Total RNA was extracted from 5th-instar nymphs at 48 h after molting, and the transcript level variation of each *NlOsiris* was analyzed via qRT‒PCR, as described in Fig. 2. The results of triplicate experiments (n = 5) are shown with standard deviations. Significant differences were calculated via Student’s *t* test (*P < 0.05 and **P < 0.01). **C** Abnormal phenotypes in *N. lugens* nymphs and adults upon *NlOsiris* gene knockdown. Group A: Abnormal body size and morphology were observed in the 5th-instar nymph (upper) and adult stages (lower panel). The ovaries were observed in female adults at 72 h after emergence. Group B: Abnormal wing phenotype. Deficient wing forms were observed in short-winged adults at 24 h after eclosion. Group C: Abnormal ovipositor phenotype. Abnormal ovipositors were observed in the adult females at 24 h after eclosion. The red arrowheads show the ovipositors in the abdomen integuments. For the RNAi experiments, each specific dsRNA (50 ng per insect) of the target genes was injected into 4th-instar nymphs, and the phenotypes were observed after RNAi. The ds*GFP*-injected insects served as controls

To determine the functions of the *Osiris* gene family, we adopted an RNAi approach to knock down the transcript levels of each *Osiris* gene in 4th-instar *N. lugens* nymphs to observe the phenotypic changes. The knockdown of 9 genes, *NlOsiris2*, *NlOsiris7*, *NlOsiris9*, *NlOsiris11*, *NlOsiris14*, *NlOsiris16*, *NlOsiris17*, *NlOsiris*19 and *NlOsiris24*, led to a significant decrease in the survival rates of each target dsRNA-injected insect (Fig. 3A). Following RNAi, more than 80% of individuals in the ds*NlOsiris14-*, ds*NlOsiris17-*, and ds*NlOsiris24-*injected groups survived at 4 days post-injection (dpi), but these percentages decreased at 5 dpi (5th-instar nymphs) and declined dramatically to approximately l0% at 13 dpi (Fig. 3A). In the ds*NlOsiris2-*, ds*NlOsiris7-*, ds*NlOsiris9-*, ds*NlOsiris16-* and ds*NlOsiris*19-injected groups and the ds*NlOsiris11-*injected group, greater than 80% of individuals successfully molted into adults at 7 dpi; however, the survival rates decreased to 50% and 30% at 13 dpi, respectively. In contrast, knockdown of the other *NlOsiris* genes, including *NlOsiris3*, *NlOsiris6*, *NlOsiris8*, *NlOsiris10*, *NlOsiris12*, *NlOsiris18*, *NlOsiris20*-*23*, resulted in greater than 70% survival rates at 13 dpi, which were not significantly different, with 85% survival rates in ds*GFP*-injected individuals at 13 dpi. To confirm the RNAi effect, we conducted qRT‒PCR analysis to investigate the variation in the transcript level of each *NlOsiris* gene in 5th-instar nymphs at 48 h after molting. The results revealed that the transcript level of each *NlOsiris* gene was significantly lower in the ds*NlOsiris*-injected nymphs than in the ds*GFP*-injected controls (Fig. 3B).

The knockdown of *NlOsiris* genes generated different morphological defects in nymphs and/or adults (Fig. 3C), which can be divided into three major types: Group A: Abnormal body size: 20–25% of ds*NlOsiris7-*, ds*NlOsiris11-* or ds*NlOsiris14-*injected nymphs showed obviously shriveled abdomens and small body sizes at the 5th-instar nymphal stage. Only 50% of these nymphs could completely transition from 5th-instar nymphs to adults but had low survival rates at the adult stage. In addition, 50% of the ds*NlOsiris2-*injected nymphs and 80% of the ds*NlOsiris11-* or ds*NlOsiris14-*injected nymphs had flat abdomens at the adult stage, whereas more than 95% of the ds*GFP*-injected controls had obviously swollen abdomens and stretched intersegmental membranes in the tergums. Notably, these adults presented deficient ovaries without mature oocytes on the 3rd day post-eclosion, whereas ds*GFP*-injected adults presented normal ovaries with banana-shaped oocytes. Group B: Abnormal wing form: 85% of the ds*NlOsiris19-*injected nymphs and 90% of the ds*NlOsiris24-*injected nymphs successfully developed into adults, but the short-winged adults presented deficient wing forms. Their forewings were very small and irregularly shaped, whereas more than 95% of the ds*GFP*-injected controls had wings of normal size and regular shape. Group C: Abnormal ovipositor: Fewer than 50% of the ds*NlOsiris17*-injected nymphs developed into adults. However, 85% of the female adults presented abnormally transparent and soft ovipositors in the abdomen integuments, whereas more than 95% of the ds*GFP*-injected females presented brown and hard ovipositors in the abdomen integuments.

### Effects of *NlOsiris* gene knockdown on feeding

**Fig. 4:**
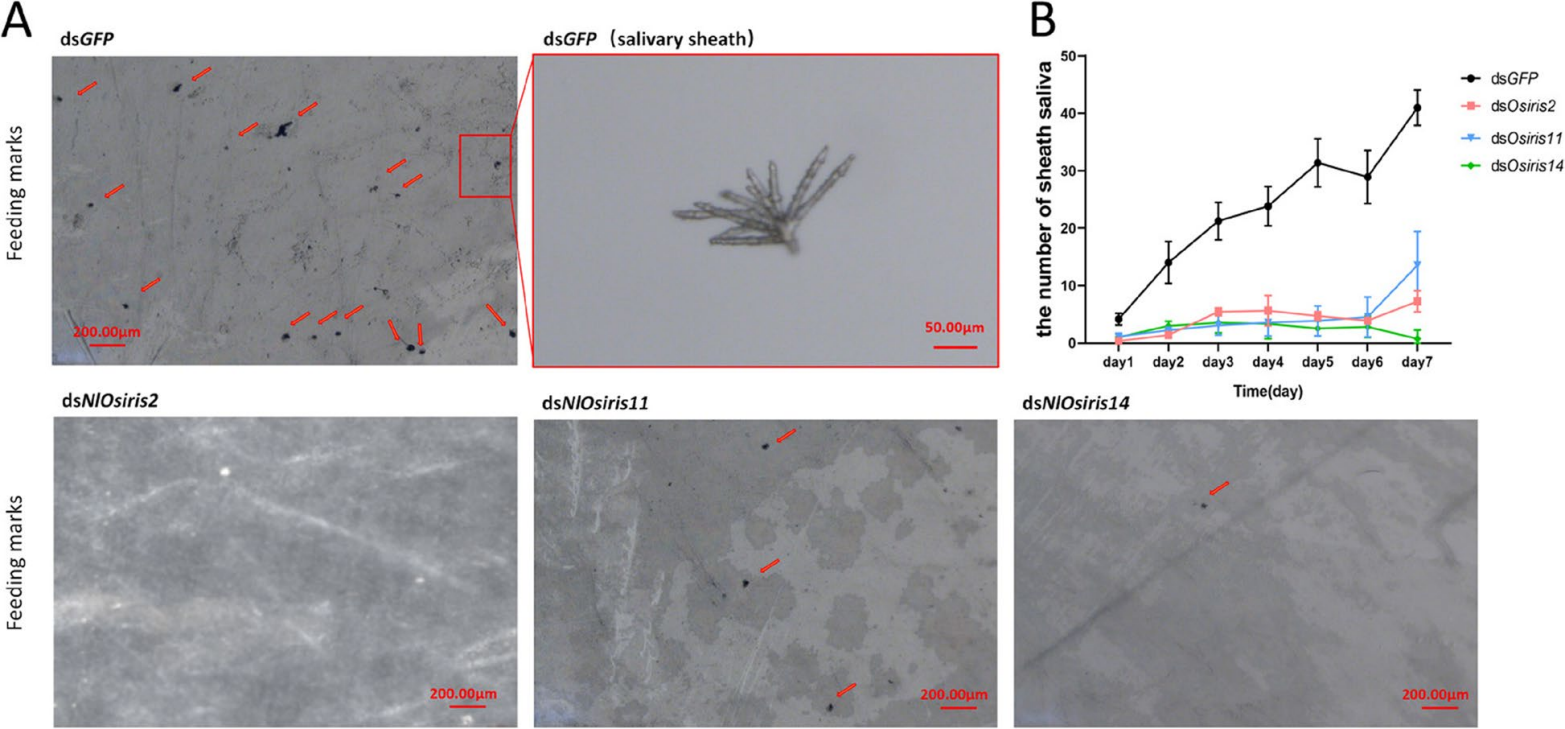
Effects of *NlOsiris* knockdown on feeding behavior. **A** Observation of salivary sheath production. The 5th-instar nymphs were injected with ds*NlOsiris2*, ds*NlOsiris11* or ds*NlOsiris14* (50 ng per insect) and were fed an artificial diet. The feeding behavior was observed, and the number of salivary sheaths on the Parafim/cm^2^ was calculated two days after injection. **B** Average number of salivary sheaths per insect. Each biological replicate consisted of 15 individuals, with a total of 4 biological replicates. The error bars indicate the standard deviations (*n* = 4)

We subsequently investigated whether flat abdomen was caused by feeding inhibition. The adults that were injected with ds*NlOsiris2*, ds*NlOsiris11* or ds*NlOsiris14* among the 4th-instar nymphs were fed an artificial diet, and their feeding behavior was observed by calculating the number of salivary sheaths in the parafilm membrane. As a result, ds*GFP*-injected adults generated more than 10 salivary sheaths/cm^2^ in the parafilm membrane on the 2nd day, which increased to 40 salivary sheaths/cm^2^ on the 7th day after feeding (Fig. 4A, B). In contrast, ds*NlOsiris2*-, ds*NlOsiris11*- or ds*NlOsiris14*-injected adults produced fewer than 10 salivary sheaths/cm^2^ in the parafilm membrane throughout 7 days of feeding (Fig. 4A, B), suggesting that *NlOsiris* knockdown inhibited feeding.

### Effects of *NlOsiris* gene knockdown on female reproduction

**Fig. 5:**
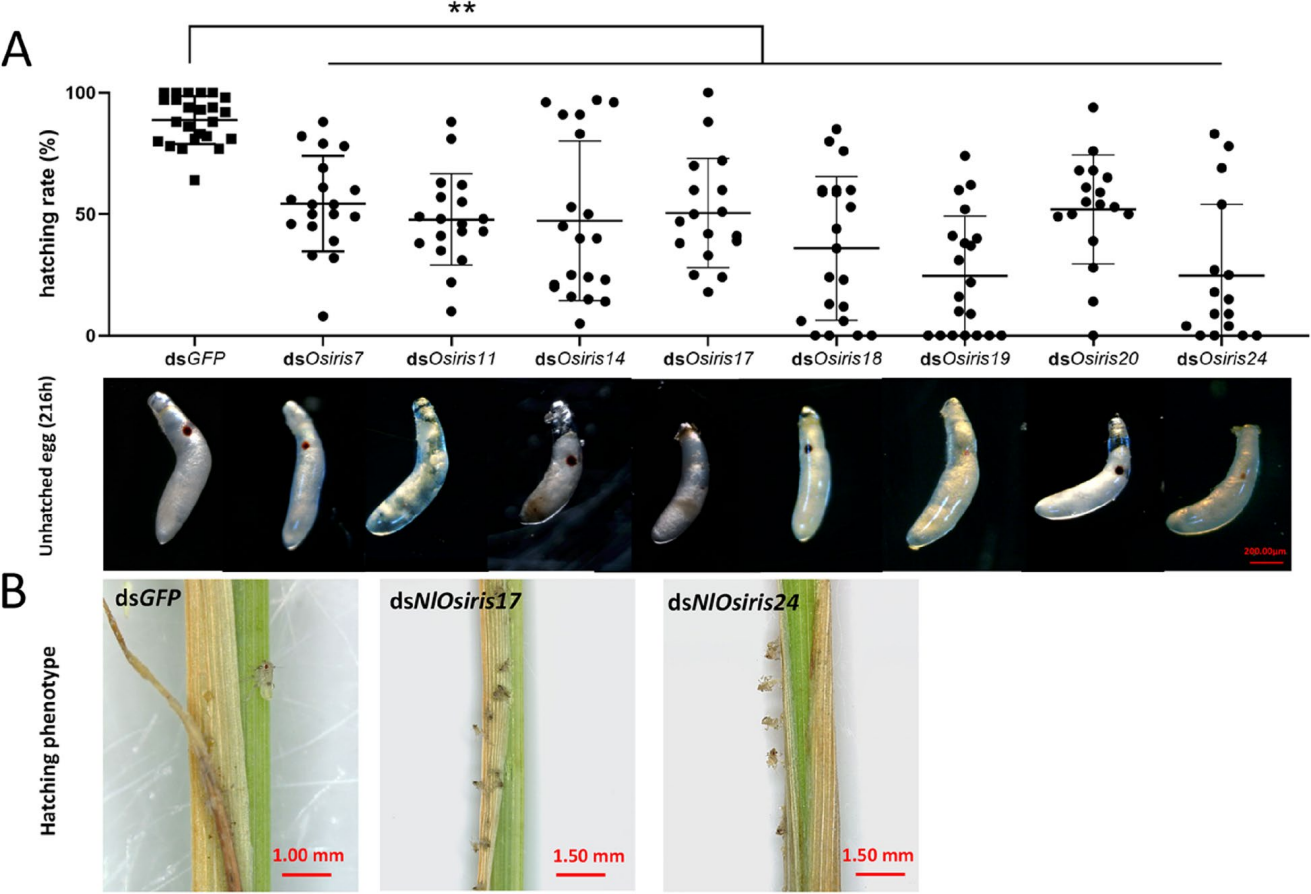
Hatching rate following the injection of dsRNA targeting the *Osiris* gene. The dsRNA of the target gene was injected into female adults 0–2 h posteclosion. **A** Injection of ds*Osiris7*, dsO*siris11*, ds*Osiris14*, ds*Osiris17*, ds*Osiris18*, ds*Osiris19*, ds*Osiris20*, or ds*Osiris24* resulted in a significant decrease in the hatching rate of fertilized eggs. The error bars indicate the standard deviations (*n* = 3). A significant difference was detected via a Student’s *t* test (*P < 0.05 and **P < 0.01). Reproductive phenotypes following the injection of dsRNA targeting the *Osiris* gene. The eggs were dissected from the rice leaf sheaths (216 h after laying). **B** After ds*Osiris17* and ds*Osiris24* were silenced, the 1st instar nymphs could not hatch normally

As most *NlOsiris* genes presented high transcript levels in eggs at 144 h after oviposition (Fig. 2A), we investigated the functions of *NlOsiris* genes in egg hatching by injecting each target dsRNA into newly emerged adults (0–2 h posteclosion). Knockdown of *NlOsiris* resulted in a significant decrease in hatching rates (Fig. 5A). Only 30–55% of the eggs laid from ds*NlOsiris7-*, ds*NlOsiris11-*, ds*NlOsiris14-*, ds*NlOsiris17-*, ds*NlOsiris18-*, ds*NlOsiris19-*, ds*NlOsiris20-* or ds*NlOsiris24-*injected female adults hatched to nymphs. In contrast, 90% of the eggs generated from ds*GFP*-injected female controls successfully hatched to nymphs. To understand the reason for hatching failure, we dissected the eggs laid in the rice leaf sheaths at 216 h, a time point just before hatching. More than 70% of the eggs from each target ds*NlOsiris-*injected female presented a typical banana shape with dark red and large eye spots. The presence of an eye spot in the egg was considered a viable fertilized egg, whereas those without an eye spot were considered unfertilized eggs [35]. Fewer than 30% of the eggs laid from ds*NlOsiris11-*, ds*NlOsiris19-* or ds*NlOsiris24-*injected females had no or only a light red and small eye spot, indicating unfertilized or abnormal eggs. Although these eggs were most likely to fail to hatch, our observations imply that the low hatching rates were probably not due to fertilization. Therefore, we observed egg–nymph transition in the rice leaf sheaths. We focused on ds*NlOsiris17-* and ds*NlOsiris24-*injected females to observe nymph hatching from the laid eggs. As a result, 60% of eggs were able to hatch into the 1st-instar nymphs; however, these insects stuck to the rice stems and could not leave, leading to hatching failure (Video S1). We confirmed the RNAi effects on the *NlOsiris7, NlOsiris11, NlOsiris14, NlOsiris17, NlOsiris18, NlOsiris19, NlOsiris20* and *NlOsiris24* genes in eggs 144 h after laying via qRT‒PCR. The transcript level of each *NlOsiris* gene was significantly lower in the ds*NlOsiris*-injected eggs than in the ds*GFP*-injected controls (Fig. S2).

## Discussion

With the completion of genomic and transcriptomic sequencing of the brown planthopper *N. lugens*, identification of the *Osiris* gene family via bioinformatics tools has become possible. Advanced technologies such as RNAi and gene editing have enabled researchers to explore the molecular functions of the *Osiris* family further. In this study, we identified and characterized 20 *N. lugens Osiris* genes by searching the *N. lugens* genomic and transcriptomic databases. The published literature indicates that insects have approximately 20 *Osiris* genes in their annotated genomes. Compared with the Diptera model insect *D. melanogaster*, *N. lugens* lacks the *Osiris1*, *Osiris4*, *Osiris5* and *Osiris13* genes, implying that these genes may play species-specific roles in *D. melanogaster*. Phylogenetic analysis revealed that each deduced *N. lugens* Osiris protein exhibited close evolutionary relationships with homologs from *D. melanogaster* and *B. mori*, suggesting a high degree of sequence conservation across diverse insect species. This conservation was evident not only in sequence homology but also in the synteny of the *Osiris* genes within their respective genomes. We analyzed the *Osiris* gene locus in the genomes of 23 insect species from eight orders. The results revealed that the synteny of the *Osiris* genes was present on one of the chromosomes of all the investigated insect genomes. The closer the evolutionary relationships are, the more similar the *Osiris* gene location and arrangement in the chromosome. *Osiris* gene clusters on one chromosome were most likely due to gene duplication during insect evolution.

**Fig. 6:**
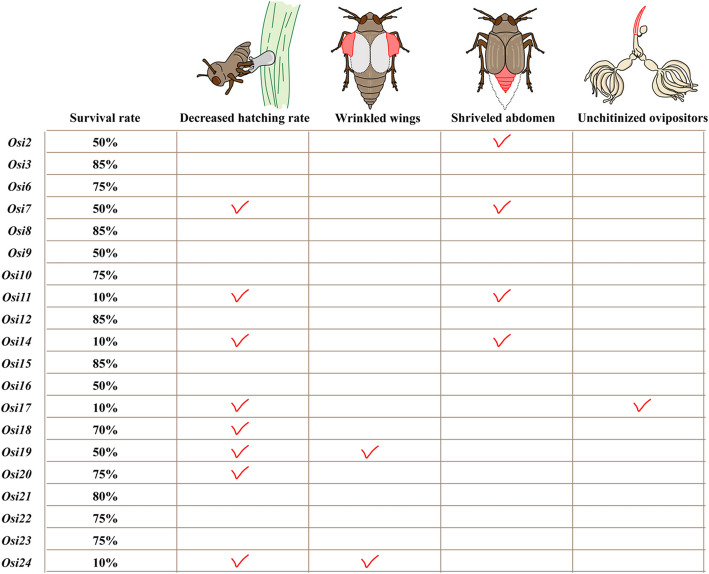
Abnormal phenotypes following the injection of dsRNA targeting each *Osiris* gene. The insects were treated as described in the Materials and Methods. Survival rate: survival rate on day 13

Temporal and spatial expression profiles revealed that *NlOsiris* genes were almost undetectable in adult stages. However, pronounced expression peaks were observed during the mid-developmental stages of each instar nymph and in the eggs at 144 h post-laying within the rice leaf sheaths. These findings align with those of previous studies [11], which reported that *Osiris* genes are crucial for epidermal development during the larval and pupal stages. Notably, our data revealed that the timing of the peak expression of *NlOsiris* genes coincided with that of chitin binding-related genes (data not shown), suggesting that *NlOsiris* genes co-expressed with chitin metabolism-related genes and might be involved in the development of the epidermis during each nymphal stage. The tissue-specific expression profiles provided further evidence that most *NlOsiris* genes were specifically expressed in the integuments and wing buds of 5th-instar nymphs, which supported our speculations about the functions of *Osiris* in the cuticle. Several studies have established a link between *Osiris* gene expression and cuticle development. In *D. melanogaster*, 7 *Osiris* genes have been implicated in tracheal maturation, while chitin plays a crucial role in the construction of the tracheal system [8]. Silencing the locust *LmOsiris17* gene resulted in abnormal development of the new cuticle [18]. Although our study did not provide direct evidence demonstrating the functional associations of *NlOsiris* genes with chitin metabolism, we observed deficient phenotypes of the cuticle morphology, which were caused by the knockdown of some *NlOsiris* genes (Fig. 6). (1) Knockdown of *NlOsiris7*, *NlOsiris11* or *NlOsiris14* resulted in significantly smaller body sizes with shriveled abdomens in 5th-instar nymphs, and knockdown of *NlOsiris2* or *NlOsiris11* or *NlOsiris14* produced thin adults with visibly flattened abdomens. (2) Knockdown of *NlOsiris19* or *NlOsiris24* resulted in abnormally small and irregularly shaped forewings in adults. (3) Knockdown of *NlOsiris17* resulted in abnormally transparent and soft ovipositors in the female abdomen integuments. These observations indicate that some *Osiris* genes have important functions in cuticle formation and structure. In addition, we found that the knockdown of *NlOsiris2*, *NlOsiris11* or *NlOsiris14* significantly affected feeding on the artificial diet. Few salivary sheaths formed at the feeding sites, which was consistent with the shriveled abdomen and decreased body weight of the *NlOsiris2-, NlOsiris11-* or *NlOsiris14*-injected insects. We hypothesize that expression silencing of these *NlOsiris* genes probably changed the mouthpart structure, thereby reducing feeding ability. Notably, the knockdown of *NlOsiris2*, *NlOsiris11* or *NlOsiris14* resulted in the generation of deficient ovaries in female adults that failed to lay eggs. These findings strongly suggest that the three *Osiris* genes have similarly important functions in feeding and female reproduction.

The transcripts of the *NlOsiris* genes were barely detectable at 24 and 72 h postlaying, corresponding to the early and middle development stages of the laid eggs. However, the transcript levels were significantly elevated in eggs at 144 h postlaying, a late developmental stage, implying that *NlOsiris* are involved in hatching. Our results revealed that the knockdown of 8 *Osiris* genes, *NlOsiris7*, *NlOsiris11*, *NlOsiris14*, *NlOsiris17*, *NlOsiris18*, *NlOsiris19*, *NlOsiris20* or *NlOsiris24*, significantly decreased the hatching rates, indicating the functional importance of these genes for hatching. In *N. lugens*, the presence of an eye spot in the egg was considered a viable fertilized egg, whereas those without an eye spot were considered unfertilized eggs [35]. In our previous study, we reported that a large and dark red eye spot appeared in typical banana-shaped eggs approximately 120 h after laying and presented along with egg development until hatching. In this study, more than 70% of the eggs laid from the above ds*NlOsiris*-injected females presented an eye spot at 216 h, a time point just before hatching, implying that most of the laid eggs were fertilized eggs. On the basis of these observations, we suppose that the reason for hatching failure was not fertilization. The subsequent experiments confirmed our speculation. We found that the knockdown of *NlOsiris17* or *NlOsiris24* intriguingly generated phenotypes of hatching failure. Most of the laid eggs have transitioned to 1st-instar nymphs; however, these nymphs were not able to hatch successfully and died in rice leaf sheaths, strongly suggesting that *NlOsiris* genes are involved in the transition of egg–nymph.

The phylogenetic tree reflected, to some extent, the functional similarities and diversities of the *Osiris* family. For example, *NlOsiris17* was closely related to *NlOsiris24*, and knockdown of its gene expression generated similar hatching failure phenotypes. Similarly, *NlOsiris18* had a close evolutionary relationship with *NlOsiris19*, with gene knockdown leading to a significant reduction in hatching rates. Interestingly, despite divergent from each other, *NlOsiris2*, *NlOsiris11*, and *NlOsiris14* presented similar RNAi phenotypes, suggesting that these genes may share common regulatory mechanisms or participate in overlapping pathways [36, 37]. In the future, it will be necessary to utilize comparative transcriptome sequencing to uncover the downstream pathways and molecular targets associated with *Osiris* gene expression, thereby providing insights into the detailed regulatory mechanisms involved.

Although the *Osiris* genes presented similar temporal‒spatial expression profiles, they presented functional diversity and specificity across insect species. For example, *B. mori Osiris9a* is specifically expressed in silk glands and is necessary for silk fiber formation [17, 38]. In contrast, knockdown of the homologous gene *Osiris9* in *N. lugens* resulted in an apparently lethal phenotype. *Drosophila melanogaster Osiris* genes are highly expressed at 48 h in the pupal stage [11]. The knockdown of *DmOsiris* genes, such as *Osiris7*, in the larval stage resulted in larvae failing to complete metamorphosis and remaining at the prepupal stage. *Nilaparvata lugens* undergoes incomplete metamorphosis, in which the nymph is essentially similar to an adult, and there is no pupal stage. Knockdown of the homologous *Osiris7* in *N. lugens* resulted in small body sizes in the nymphs and decreased hatching rates, suggesting that the functions of the homologous *Osiris* genes differ between holometabolous and hemimetabolous insect species.

*Osiris* genes densely clustered on one of the chromosomes across insect species. Synteny-generated genetic redundancy has been reported for Drosophila *Osiris9*, *Osiris15*, and *Osiris19* in tracheal function [8]. In this study, the knockdown of nine *NlOsiris* genes, e.g., *Osiris3*, *Osiris6*, *Osiris8*, *Osiris10*, *Osiris12*, *Osiris15*, and *Osiris21-23*, did not cause notable changes at the individual or tissue level, such as changes in survival rates, hatching rates or cuticle morphology. However, this does not imply that they are functionally unimportant, as some *NlOsiris* genes may have redundant roles owing to their similar development-specific and tissue-specific expression patterns.

In conclusion, *Osiris* genes play crucial roles in wing, mouthpart, ovipositor and ovary development, as well as in egg hatching and feeding, ensuring survival and reproductive success. Our study provides insights into the physiological functions of *Osiris* and highlights their potential association with cuticle development. Further studies are needed to clarify the precise regulation and action mechanisms of the *Osiris* gene during insect development and reproductive processes, e.g., to elucidate the relationships between the *Osiris* genes and chitin metabolism in insect cuticles. Further studies should focus on determining the specific functional roles and interactions of *Osiris* gene family members via CRISPR/Cas9 gene editing in insects.

## Supplementary Information

Supplementary Material 1.

Supplementary Material 2.

Supplementary Material 3.

Supplementary Material 4.

Supplementary Material 5.

## Data Availability

The datasets and materials supporting the results are included within the article, and the work described has not been published before.
