# Supplementary figures and images for: Functional diversity of the Osiris gene family in the brown planthopper
Source: Crop Health. 2025 Mar 12;3(1):7. doi: 10.1007/s44297-025-00045-4 (PMC12825958; doi:10.1007/s44297-025-00045-4)

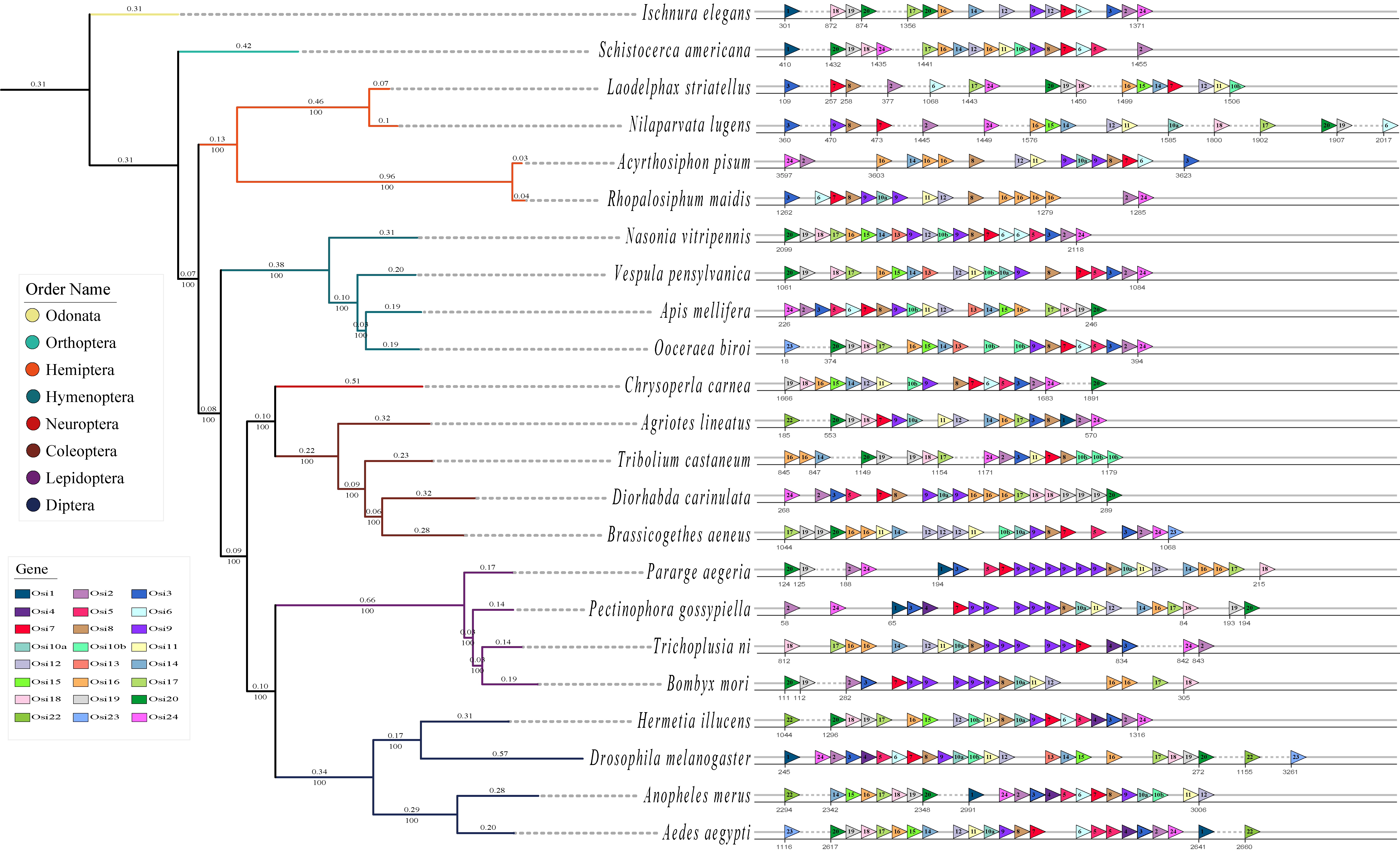

Supplement: Supplementary file 1 — Supplementary Material 1. [file 44297_2025_45_MOESM1_ESM.png]

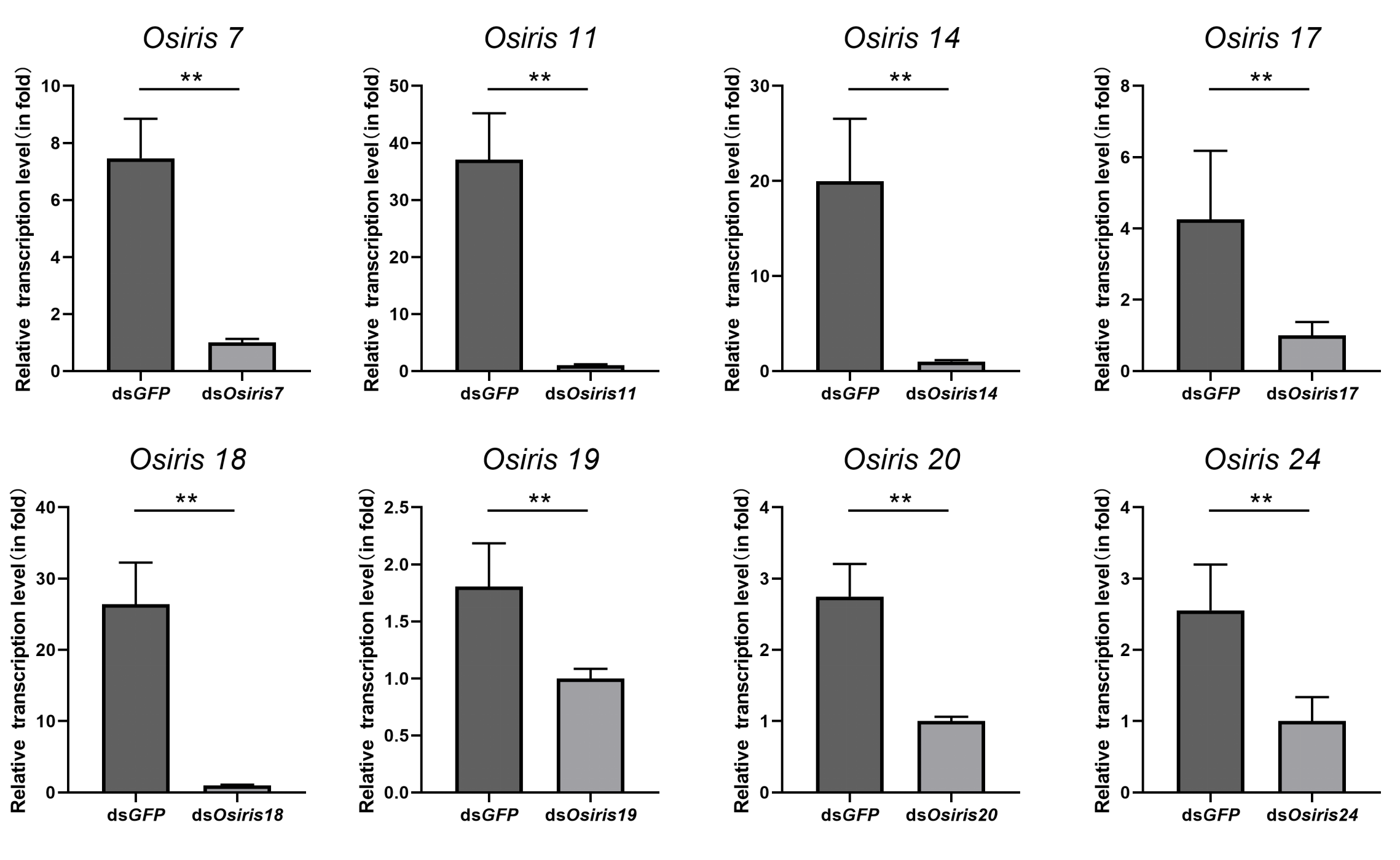

Supplement: Supplementary file 2 — Supplementary Material 2. [file 44297_2025_45_MOESM2_ESM.png]
