# Supplementary material for: Functional diversity of the Osiris gene family in the brown planthopper
Source: Crop Health. 2025 Mar 12;3(1):7. doi: 10.1007/s44297-025-00045-4 (PMC12825958; doi:10.1007/s44297-025-00045-4)
Supplement: Supplementary file 3 — Supplementary Material 3. [file 44297_2025_45_MOESM3_ESM.docx]

|  | T7-F | T7-R | Qrt-F | Qrt-R |
| --- | --- | --- | --- | --- |
| *osiris 2* | TTGCCGTCAGTGAACATC | GCTTCTTGAACTTGGTCCTA | TTGCCGTCAGTGAACATC | AATCGCCTTCCAGACACA |
| *osiris 3* | AGAAGGTCAAGGACGAGAA | CAACAGGAACGCCAACTT | GGTATGATGAAGATCGGAATG | TGAGTCACCACCTCGTAG |
| *osiris 6* | TCTGTTCGCTGGATTGTC | CCACATTGTTGGAGTAGGAT | CGAGAAGAAGGACGATGTT | CAGACCAATCAGGAGAAGAG |
| *osiris 7* | GTGACAGTGGTGAGGAAC | GAGCGTATGCGTTGTAGG | GGCAGTAGTATTGTGGTCTT | GTCGCTGTAGATGCTGTC |
| *osiris 8* | CATCGTAGCCAACAACATC | CCACCAGCTTCTTGAGTC | GACAACATCGTAGCCAACA | CCGCAGAATCCAGTGTAG |
| *osiris 9* | TGCTGTCGGTAGTTGAATC | GACGATGAGTGCCTTGAG | TGCTGTCGGTAGTTGAATC | AAGTCCTTGCCACCACAT |
| *osiris 10* | AGTCGTTGTTGGTGCTTAT | TGGAATTATGGCGATCAGAT | CCTGATATTCCTCACCAAGAA | GCGATACAGACTGCCATC |
| *osiris 11* | GACCAGGACGACACTATG | GATTGGCTGCTTCACGAT | ATTGAAGGAGTATCGTTGGT | CTTGGTCAGTGGAGTCATC |
| *osiris 12* | ATGTGGCTGAAGTGGTGT | CGATCTTGCTGATGATGAAC | CACCGAGAACGACTTGGA | GCGACCTTCTTCAACACTC |
| *osiris 14* | GAGAGCATCTCTGTCATCC | GGCACTTGAGCATTGAAC | ATCCTCTCGCCTTGTTGA | AACCAGTCCAATGATTCCAA |
| *osiris 15* | GGACTGAGTGAGGAGGAA | CGAAGAATCAAGCCAAGTG | ATGTTGTTCTTGGTGGTGTA | ATGCCTTCTGAGCGTAAC |
| *osiris 16* | GACACCGATTTCCAGATTCT | GTAGCCGTAACCACTGTG | GAGCACTGATACTGACAATAC | GCTTGGTGATGATCTCGTA |
| *osiris 17* | AGACTTCTATGTGACTGATGG | TGGCTCTATGTTGAACTTGA | AGACTTCTATGTGACTGATGG | TTCTCCTCTTCCACCTTGT |
| *osiris 18* | GAGACAGCATCGGACATC | TCGTGGAAGTTGTGAATCT | TCCTGATTGGTCTGAAGTTC | GTGGAAGTGCTCGTAGTT |
| *osiris 19* | CAGTAAGACACCAGCAGTC | CGAACGAGAAGAAGGAGAG | CAGTCTGTGCTCTGTTCC | AGATACCTGAATAGTGTCCTTG |
| *osiris 20* | CTCGTGTCTTGTGCTTGT | ACTCCGCCATCATCATCT | GATGATGATGGCGGAGTC | GGCTGTTGGATTGAGAGTT |
| *osiris 21* | CGACTACCGACTCCTCAT | GGCATAAGCACTGTAGGC | TGTGTCCTTCGTCCTCTT | GTAGTTGGCATCGTTGGT |
| *osiris 22* | GAACGAGGTACTACTCTACTG | CATCATATCCGTAATGCTCCT | CCTTCCACTGTTGCTCAT | TCATATCCGTAATGCTCCTT |
| *osiris 23* | CCAACAATGAACAGGTCAAC | GACAACAGCTTCTTGAATCC | CCAAGATAACAGCACAGATG | ATCACTGAGCGGTATGTAG |
| *osiris 24* | GCCAGCAATCTGATAGTGA | CAGTGTTGGTTGTGTCCTT | TACTGCCGCTGTTCATTG | GTGTTGGTTGTGTCCTTGA |
|  |  |  |  |  |
| T7 promoter | TAATACGACTCACTATAGGGAGA |  |  |  |

**Table S1** Primers of the *Osiris* gene family in *N. lugens*
